# Tuberculosis Surveillance and Control, Puerto Rico, 1898–2015

**DOI:** 10.3201/eid2503.181157

**Published:** 2019-03

**Authors:** Emilio Dirlikov, Dana Thomas, David Yost, Betzaida Tejada-Vera, Maria Bermudez, Olga Joglar, Terence Chorba

**Affiliations:** Centers for Disease Control and Prevention, Atlanta, Georgia, USA (E. Dirlikov, D. Thomas, D. Yost, O. Joglar, T. Chorba);; United States Public Health Service, Commissioned Corps, Rockville, Maryland, USA (D. Thomas, D. Yost);; National Center for Health Statistics, Hyattsville, Maryland, USA (B. Tejada-Vera);; Puerto Rico Department of Health, San Juan, Puerto Rico (M. Bermudez, O. Joglar)

**Keywords:** Tuberculosis, TB, Puerto Rico, elimination, history, Caribbean, tuberculosis and other mycobacteria, bacteria, surveillance, United States

## Abstract

The World Health Organization recognizes Puerto Rico as an area of low tuberculosis (TB) incidence, where TB elimination is possible by 2035. To clarify the current low incidence of reported cases, provide key lessons learned, and detect areas that may affect progress, we systematically reviewed the literature about the history of TB surveillance and control in Puerto Rico and supplemented this information with additional references and epidemiologic data. We reviewed 3 periods: 1898–1946 (public health efforts before the advent of TB chemotherapy); 1947–1992 (control and surveillance after the introduction of TB chemotherapy); and 1993–2015 (expanded TB control and surveillance). Although sustained surveillance, continued care, and use of newly developed strategies occurred concomitantly with decreased reported TB incidence and mortality rates, factors that may affect progress remain poorly understood and include potential delayed diagnosis and underreporting, the effects of government debt and Hurricane Maria, and poverty.

Tuberculosis (TB) remains a major public health challenge. Worldwide in 2017, an estimated 10 million incident cases occurred (*1*); ≈1.3 million deaths were caused by TB, and another 300,000 deaths were caused by TB among HIV-positive persons. TB now ranks above HIV as the leading cause of death from an infectious disease (*1*). On September 26, 2018, the United Nations held its first high-level meeting on TB to accelerate efforts to end TB by reaching all affected persons with prevention and care.

Progress has been made toward global targets since the World Health Organization (WHO) declared a Global TB Emergency in 1993 (*2*). WHO has recognized 33 low-incidence countries and territories where the goal of TB elimination, defined as <1 case/1 million population, is possible by 2035 (*3*). Puerto Rico, a US territory, is one of the low-incidence areas. During 1993–2015, incidence of reported cases decreased from 7.1 to 1.5 cases/100,000 population (*4*). Still, challenges to surveillance and control remain.

To learn more about decreasing incidence of reported TB cases and mortality rates, provide key lessons learned, and discuss areas that may affect progress toward TB elimination, we reviewed the history of TB surveillance and control in Puerto Rico. We divided our review into 3 periods (Table 1; Figure 1): 1898–1946 (public health efforts before the advent of TB chemotherapy); 1947–1992 (control and surveillance after the introduction of TB chemotherapy, which in Puerto Rico was predominantly delivered through government-subsidized primary health services); and 1993–2015 (expanded TB control and surveillance, which followed renewed efforts in the United States to control TB and the introduction of Reforma de Salud de Puerto Rico, a government-run healthcare program managed through private insurance companies). 

**Table 1:** Summary of the evolution of TB surveillance, diagnosis, and treatment in Puerto Rico over 3 periods*

Period	Surveillance	Diagnosis	Treatment
1898–1946	Passive, relying on voluntary reporting; Bureau of TB established in 1924; TB becomes a part of vital statistics centrally compiled by PRDH in 1931	Primarily clinical diagnosis; TST progressively routinized after 1929; chest radiography and limited sputum examination in TB dispensaries since 1935	Pneumothorax procedures, bed rest in sanatorium, and isolation of patients with active TB
1947–1992	TB recording and reporting through PRDH TB centers and centralized at PRDH; private physicians, hospitals, and VA report to PRDH; case-level data collection and reporting to NTSS uses RVCT	TST and chest radiography routine for screening (e.g., medical cards); limited sputum AFB examination and ability to culture	Three-drug regimen of streptomycin, PAS, and isoniazid; free treatment in 3-mo courses for total treatment of >2 y; introduction of short-course regimens using rifampin in the 1970s
1993–2015	RVCT revised and expanded; CDC NTSS electronic registry launched; molecular testing for DST at PRDH laboratory and genotyping and molecular testing through CDC introduced	Diagnosis relies on sputum AFB examination and culture; screening with TST and chest radiography; few cases diagnosed clinically	DOT, 4-drug regimen of isoniazid, rifampin, pyrazinamide, and ethambutol; intensive and continuation phase for 6–8 mo; regimens individualized according to DST results

*AFB, acid-fast bacillus; CDC, Centers for Disease Control and Prevention; DOT, directly observed therapy; DST, drug-sensitivity testing; NTSS, National TB Surveillance System; PAS, para-aminosalicylic acid; PRDH, Puerto Rico Department of Health; RVCT, Report of Verified Case of Tuberculosis; TB, tuberculosis; TST, tuberculin skin testing; VA, Veterans Administration.

**Figure 1:**
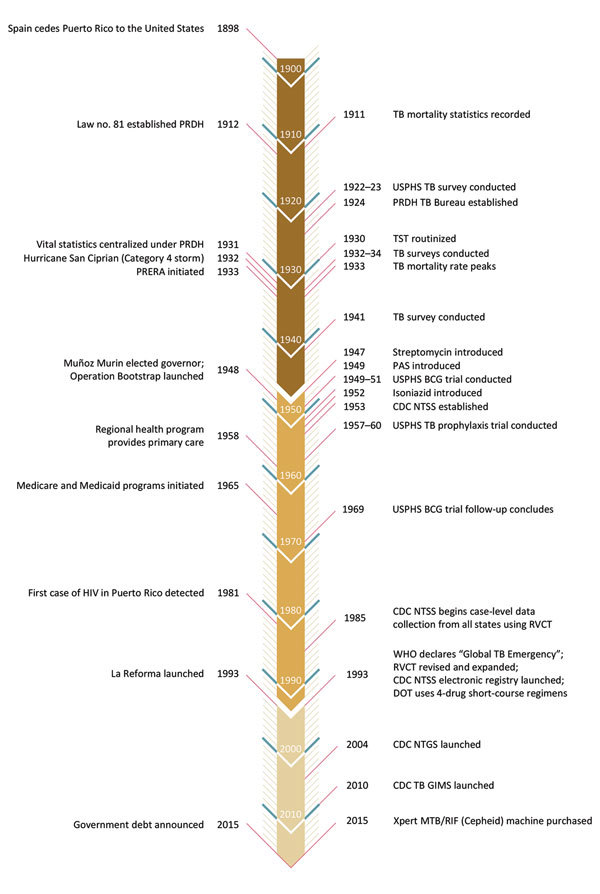
Timeline of key TB control events in Puerto Rico (right) and developments in healthcare and politics (left), over 3 periods: 1898–1946, 1947–1992, and 1993–2015. BCG, bacillus Calmette-Guérin vaccine; CDC, US Centers for Disease Control and Prevention; DOT, directly observed therapy; La Reforma, Reforma de Salud de Puerto Rico; PAS, para-aminosalicylic acid; NTGS, National TB Genotyping Service; NTSS, National TB Surveillance System; PRDH, Puerto Rico Department of Health; PRERA, Puerto Rico Emergency Relief Administration; RVCT, report of verified case of tuberculosis; TB, tuberculosis; TB GIMS, TB Genotyping Information Management System; TST, tuberculin skin testing; USPHS, United States Public Health Service; Xpert MTB/RIF machine, multidrug/rifampin resistance machine (Cepheid, http://www.cepheid.com).

## Study Design

### Literature Review

On August 30, 2015, we searched publications on PubMed and the US Centers for Disease Control and Prevention (CDC) Primo (CDC-based electronic literature search engine that enables users to search for books, journal articles, CDC stacks, and digital objects) for the terms “Puerto Rico” and “tuberculosis.” The search returned 240 articles, 18 of which we downloaded after reviewing titles and abstracts. Additional articles were added after a review of the article references (n = 28) and Spanish-language articles (n = 8), contextual literature (n = 26), and co-author literature databases (n = 4). We fully reviewed 84 articles and excluded 8 because of lack of relevance.

### Epidemiologic and Population Data

Epidemiologic data were primarily sourced from the CDC TB annual reports for 1953–1992 (https://www.cdc.gov/tb/statistics/reports/default.htm) and CDC Online Tuberculosis Information System (OTIS; https://wonder.cdc.gov/tb.html) for 1993–2015. We calculated mortality rates for Puerto Rico and the United States (excluding Puerto Rico) by using epidemiologic and population data sourced from the following databases: CDC WONDER (https://wonder.cdc.gov/mortSQL.html), Vital Statistics (https://www.cdc.gov/nchs/nvss/mortality_historical_data.htm), US Census Bureau (https://www.census.gov), and the United Nations Statistics Division (https://unstats.un.org) (Appendix).

### Statistical Analyses

Using reported TB cases from 1993–1997 as a referent, we analyzed patient age distribution for reported TB cases from 2011–2015 by using the χ^2^ test. We considered p<0.05 to be significant.

## Results

### Public Health Efforts before the Advent of TB Chemotherapy (1898–1946)

In 1898, Spain ceded Puerto Rico to the United States through the Treaty of Paris. Thereafter, health directives were implemented under US territorial administration. Building on nascent efforts, on March 14, 1912, law no. 81 created the Puerto Rico Department of Health (PRDH), charged with protecting public health on the island.

The annual reports of the Puerto Rico Commissioner of Health TB statistics included mortality rates. TB notification relied on voluntary reporting from private physicians and healthcare centers across the island. During the 1920s, the PRDH collected additional health-related data and compiled statistics, including those for TB.

**Figure 2:**
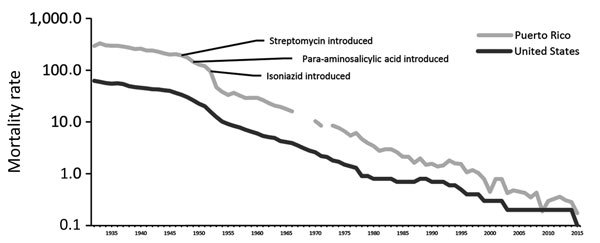
Mortality rate (no. deaths/100,000 population, on logarithmic scale) for reported TB cases in Puerto Rico and the United States, 1932–2015. Mortality data for Puerto Rico were not recorded for 1967–1969 and 1972.

During 1922–1923, the US Public Health Service (USPHS) conducted the first TB survey of Puerto Rico by using records from schools, the US Veteran’s Bureau, Army enlistments, private practitioners, and hospitals (*5*). The USPHS concluded that estimating TB morbidity rates was not possible because of underreporting; an estimated 60% of deaths from TB were not recorded (*5*,*6*). In 1924, PRDH established a Bureau of Tuberculosis, which was responsible for recording TB statistics (*7*). By 1931, a centralized vital statistics system was implemented (*8*), and starting in 1932, Puerto Rico data were included in US annual mortality statistics (*9*). Recorded TB mortality rates subsequently increased, in part as an artifact of improved reporting, and peaked in 1933 at 333 deaths/100,000 population; the high mortality rate was linked in part to the destruction wrought by Hurricane San Ciprian, a Category 4 storm that traversed the island in September 1932 (*8*). By comparison, estimated TB deaths for that year in the United States were 56.7/100,000 population (Figure 2). Sanatoria, including 1 sanitarium each in Rio Piedras (200 beds) and Ponce (30 beds), were unable to cope with the high volume of patients (*10*).

In addition to regular recording and reporting activities, several TB-related surveys were conducted, leading to public health action. For example, after a 1930 survey of 2,500 school children 8–10 years of age found that 68% had positive reactions to tuberculin skin testing (TST) (*6*,*8*), TST was used in epidemiologic studies and to screen children and adolescents. Additional surveys took 1 of 2 forms. First, evaluations of postmortem and pathology specimens characterized TB among the dead, finding a “large measure of tuberculization of the people” (*11*,*12*). Second, epidemiologic surveys were conducted in a variety of settings, including coastal and mountainous municipalities (*6*), urban (*13*) and rural (*14*) communities, and low-resource communities (*15*). Surveys made use of TST results, chest radiographs, patient histories, and limited bacteriologic examinations. Several institutions were involved in conducting the surveys, including PRDH, the School of Tropical Medicine of Puerto Rico, Columbia University (New York, NY, USA), and the Rockefeller Foundation and its International Health Board (New York).

The resulting picture of TB on the island was that it primarily affected those 20–40 years of age, nonwhite more than white persons (*16*), urban more than rural areas (*7*), and women more than men (*7*). The most salient underlying factor was poverty, as evidenced by overcrowding, poor living conditions, and malnutrition (*6*,*7*). These findings combined with the high death rate led the Puerto Rico Commissioner of Health (Eduardo Garrido Morales) to prioritize TB. Drawing on grants from the Puerto Rico Emergency Relief Administration (established in 1933), government-run TB dispensaries were established and well-ventilated subsidized housing for the poor was constructed (*8*,*14*). By 1935, of 20 planned TB dispensaries, 4 were operational and outfitted to conduct fluoroscopy, chest radiography, and pneumothorax procedures (treatment by surgically collapsing infected lobes) for patients with active TB; 2 traveling clinics provided services to rural areas (*17*). Culture testing was reserved for special cases (*8*). Patients could be isolated in the TB dispensary for 15 days if they were suspected of having “open,” or active, TB (*8*).

### Control and Surveillance after the Introduction of TB Chemotherapy (1947–1992)

In 1947, the US Congress allowed Puerto Rico to elect a governor, and the next year, Luis Muñoz Marín was elected. In that same year, to accelerate industrialization and development on the island, the US federal government launched Operation Bootstrap (Operación Manos a la Obra). This operation included projects to improve health; throughout the 1950s, government-subsidized primary healthcare was scaled up and reached islandwide coverage in 1958. In 1965, Medicare and Medicaid programs were initiated on the island.

In 1953, the CDC National Tuberculosis Surveillance System was established. TB remained a major public health challenge in Puerto Rico, accounting for 128 deaths/100,000 population in 1950, a TB mortality rate 5.7 times more than that on the US mainland (Figure 2). TB control, as reflected by the accelerated decline in annual TB mortality rates, was improved by availability of free pharmaceuticals on the island, including streptomycin (introduced in 1947), para-aminosalicylic acid (1949), and isoniazid (1952) (*18*,*19*) (Figure 2). For example, the reported mortality rate fell from 128.4 deaths/100,000 population in 1950 to 10.4 deaths/100,000 population in 1970 (Figure 2). Epidemiologic data revealed that TB continued to affect predominantly those 20–40 years of age but that men were affected more than women. Along with the expanded use of chest radiography for case finding (*18*), TST was used for screening and was required before one could obtain a worker’s health certificate. During 1949–1950, PRDH took 234,487 chest radiographs, by which 1,346 (0.6%) cases of active TB were identified. Sputum examination by acid-fast bacilli or culture remained limited or of poor quality (*18*).

Ambulatory treatment was provided, and medications were disbursed in 3-month courses; total treatment duration was >2 years (*18*). Subsequent short-course regimens using rifampin were introduced in the 1970s. In-patient care was also managed at an 800-bed TB hospital, which opened in Río Piedras in 1952, and at the US Veterans Administration Hospital in San Juan. In 1958, PRDH TB centers had registered 22,000 patients with active and latent cases; 7,800 were receiving treatment (*18*). An estimated additional 2,600 patients were managed by public and private TB hospitals.

Puerto Rico was the site of 2 major USPHS TB investigations. First, during September 1949–May 1951, the USPHS collaborated with PRDH and the Puerto Rico Department of Education to conduct a controlled trial of bacillus Calmette-Guérin vaccine (*20*–*22*). During this trial, 191,827 children (1–18 years of age) in rural and urban areas were screened for TB; 50,634 (26%) of them received the vaccine, and they were compared against 27,238 (14%) controls (*23*–*25*). Follow-up continued through June 30, 1969. Although the vaccine conferred some benefit to younger persons, it had no longstanding protective effect. Second, during 1957–1960, the USPHS conducted a controlled trial of 1-year treatment with isoniazid prophylaxis (5mg/kg/d), which included 25,000 known TB patient contacts in the United States, Mexico, and Puerto Rico, 12,000 of whom were in Puerto Rico. Contacts were randomized to receive treatment drug or placebo; among those receiving isoniazid, incidence of active TB disease was 60% lower over the course of the trial (*26*,*27*). The Puerto Rico principal study investigators (José Sifontes and Carlos Vicéns) highlighted the value of prophylactic treatment: “A large reservoir of tuberculosis cases for the next generation is already seeded in Puerto Rico…. The infant with a positive tuberculin in 1962 may be the grandfather who will develop cavitary disease and infect his grandchildren in the year of 2012” (*26*). In tandem, the 2 USPHS TB investigations addressed the international debate about effective TB prevention strategies (bacillus Calmette-Guérin vaccination vs. isoniazid treatment of latent TB), and findings supported isoniazid treatment as being more effective (*25*,*28*).

During the late 1970s and early 1980s, TB funding was reduced nationally, and several PRDH TB clinics were closed (*29*). Studies found incomplete TB reporting (*30*), and decreased support for TB surveillance and control resulted in underestimation of the true population burden of TB (*29*,*31*).

### Expanded TB Control and Surveillance (1993–2015)

In 1993, health system reforms were initiated to reduce bureaucracy and increase efficiency. Reforma de Salud de Puerto Rico, locally referred to as La Reforma, was introduced as a government-run healthcare program managed through private insurance companies. Under La Reforma, TB patients could access TB diagnosis and treatment through private healthcare providers, while PRDH continues to provide care at TB clinics (*32*) and retains responsibility for laboratory services, outbreak investigations, determining when cases have had sufficient treatment to be closed, and quarantine.

Since the 1980s, TB care on the island, as elsewhere, has been complicated by 2 factors. First, the emergence of HIV resulted in TB co-infections (*33*). In Puerto Rico, compromised immunity caused by HIV infection was first recognized in 1981, and in 2015, Puerto Rico ranked among the top 10 US jurisdictions in terms of rates of HIV infection diagnoses (*34*). During 1993–2015, a total of 838 (38%) cases of HIV infection were identified among 2,232 TB patients with reported HIV testing results; an additional 930 TB patients did not have reported HIV results. During this same period, of 189,222 TB patients in the United States with reported HIV testing results, 33,227 (18%) were HIV positive.

Second, the proliferation of drug-resistant forms of TB, including multidrug-resistant (MDR) TB (defined as resistance to both isoniazid and rifampin), required new diagnostics to conduct drug-susceptibility testing (DST) and guide appropriate treatment algorithms. In the 1990s, DST was rarely conducted (*32*), although CDC recommended and financially supported PRDH to send isolates for DST to the CDC mycobacteriology laboratory in Atlanta, such as happened during an investigation of nosocomial transmission in an HIV care unit (*35*). More recently, since 2015, detection of TB drug resistance was facilitated by the purchase of an Xpert MTB/RIF machine (Cepheid, http://www.cepheid.com), housed within the PRDH TB laboratory. During 1993–2015, a total of 44 cases of MDR TB were detected, of which 24 (55%) were reported during 1993–1997.

In the early 1990s, the identification of HIV/TB co-infections and drug-resistant TB, along with an overall increase in TB patients in United States, reinvigorated national control and surveillance efforts. In 1993, the National Tuberculosis Surveillance System, which began collecting case-level data from all states in 1985 by using the Report of Verified Case of Tuberculosis (https://www.cdc.gov/tb/programs/rvct/default.htm), expanded to include HIV status, initial drug regimen, DST results, use of directly observed therapy, and completion of therapy, with reporting facilitated by an electronic registry. Furthermore, CDC has steadily introduced molecular surveillance methods (*36*–*39*). In Puerto Rico, TB genotyping has assisted epidemiologic investigations, especially within healthcare facilities (*35*,*40*–*42*). For example, during April 1993–April 1995, genotyping isolates from 113 culture-positive TB patients identified 8 clusters and helped demonstrate transmission within an AIDS care facility (*41*). In 2004, CDC established the National Tuberculosis Genotyping Service to provide genotyping services through molecular evaluation of culture-positive isolates (*43*). In 2010, CDC launched the TB Genotyping Information Management System to manage and analyze molecular epidemiologic data, including rapid detection of genotype clusters appearing across state and territorial boundaries (*44*). Starting in 2018, in the United States and its territories, whole-genome sequencing has been performed on all new isolates of *Mycobacterium tuberculosis* referred for genotyping. Use of whole-genome sequencing will further assist the PRDH TB Control Program with cluster identification and investigation and with surveillance of molecular determinants associated with drug resistance.

### Current Epidemiology and Challenges

**Figure 3:**
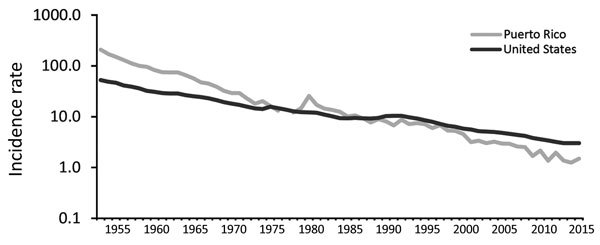
Incidence (no. cases/100,000 population, on logarithmic scale) of reported TB cases in Puerto Rico and the United States, 1953–2015. Puerto Rico data for 1953–1992 sourced from US Centers for Disease Control and Prevention annual TB reports; data not available for 1977; data for 1993–2015 sourced from the Centers for Disease Control and Prevention Online Tuberculosis Information System (https://wonder.cdc.gov/tb.html).

In 2015, PRDH reported 52 cases of TB, substantially fewer than the 274 cases reported in 1994. During 1993–2015, incidence of reported cases decreased from 7.1 to 1.5 cases/100,000 population; since the late 1980s, this rate has been lower than that of the United States (Figure 3). Of 266 TB cases reported during 2011–2015 in Puerto Rico, 188 (71%) were in male patients and 44 (17%) were in foreign-born patients; 225 (85%) had positive culture results, 14 (5%) met the clinical definition, and 26 (10%) were diagnosed by providers (https://www.cdc.gov/tb/programs/rvct/default.htm) (Table 2). Compared with the 1,272 case-patients reported during 1993–1997, case-patients reported during 2011–2015 were older (p<0.001), and a larger proportion received directly observed therapy only (134/266 [50%] during 2011–2015 vs. 252/1,272 [20%] during 1993–1997) and completed therapy within 1 year (140/266 [53%] during 2011–2015 vs. 604/1,272 [47%] during 1993–1997) (Table 2). Of the 266 TB case-patients reported during 2011–2015, culture results were positive for 225. Of those, 209 (93%) isolates underwent genotyping; half (105/209 [50%]) had unique genotypes. Of patients with positive cultures, 217 (96%) isolates underwent initial DST, and 6 (3%) cases of MDR TB were identified. Of the 245 TB patients with known HIV status, 46 (19%) were HIV positive. As of June 2018, of the 266 patients, 187 (70%) had completed treatment, 6 (2%) refused treatment, 48 (18%) died during treatment, 6 (2%) moved out of the country, 5 (2%) did not complete follow-up monitoring, and 14 (5%) had unknown outcomes.

**Table 2:** Patient demographics, verification criteria, multidrug-resistant tuberculosis results, HIV status, receipt of directly observed therapy, and therapy completion in <1 y for reported tuberculosis case cohorts, 1993–1997 and 2011–2015*

Variable	1,272 Reported cases, 1993–1997 no. (%) patients	266 Reported cases, 2011–2015 no. (%) patients
Age, y		
0–4	39 (3)	0
5–14	10 (1)	0
15–24	60 (5)	13 (5)
25–44	449 (35)	66 (25)
45–64	347 (27)	112 (42)
>65	278 (22)	73 (27)
Not available	89 (7)	2 (1)
M	869 (68)	188 (71)
F	403 (32)	78 (29)
Origin of birth		
United States	1,210 (95)	222 (83)
Other than United States	58 (5)	44 (17)
Not available	4 (<1)	0 (0)
Verification criteria		
Positive culture	1,090 (86)	225 (85)
Clinical case definition	82 (6)	14 (5)
Provider diagnosis	70 (6)	26 (10)
Positive smear/tissue	30 (2)	0 ()
Not available	0 ()	1 (<1)
Multidrug-resistant tuberculosis		
Yes	24 (2)	6 (2)
No	753 (59)	210 (79)
Not applicable or available	495 (39)	50 (19)
HIV status		
Positive	386 (30)	46 (17)
Negative	292 (23)	199 (75)
Not reported	594 (47)	21 (8)
Directly observed therapy		
Direct only	253 (20)	134 (50)
Self only	807 (63)	63 (24)
Both	25 (2)	5 (2)
Not applicable or available	187 (15)	64 (24)
Therapy completed in <1 y		
Yes	604 (47)	140 (53)
No	113 (9)	6 (2)
Not applicable or not available	555 (44)	120 (45)

Compared with other US jurisdictions, the low incidence of reported cases in Puerto Rico may result in part from delayed diagnosis and underreporting, as suggested by comparing cases reported during 2011–2015 in Puerto Rico and the United States. The lower proportion of TB cases meeting the clinical case definition in Puerto Rico (5%, 14/266) than in the United States (16%, 7,786/48,955) may denote insufficient clinical awareness of TB symptoms; the higher proportion of culture-positive cases (Puerto Rico 85%, 225/266, vs. United States 77%, 37,723/48,955) may indicate delayed diagnosis because the likelihood of detecting TB through culture increases as disease advances over time. The proportion of TB cases identified after death was also higher in Puerto Rico (5%, 14/266) than in the United States (2%, 1,065/48,955). Puerto Rico has a smaller proportion of foreign-born TB patients (17%, 44/266) than does the United States (65%, 31,764/48,955). Foreign-born patients also differed by country of origin: during 2011–2015, a total of 75% (33/44) of foreign-born Puerto Rico patients reported were from the Dominican Republic; in the United States in 2015, the top 5 countries of origin were Mexico, the Philippines, India, Vietnam, and China (*45*). Given the estimated TB incidence of 45 cases/100,000 population in the Dominican Republic in 2017 (*1*) and the high rates of documented and undocumented immigration to Puerto Rico, the extent of TB among residents originally from the Dominican Republic is poorly understood.

Socioeconomic factors and determinants of TB may also affect progress toward TB elimination in Puerto Rico (*46*). TB surveillance and control may be further complicated by the effect of the government economic debt, which was estimated at $72 billion in June 2015, and of Hurricane Maria, which made landfall on September 20, 2017, as a Category 4 hurricane. Many health professionals have left in the wake of these crises (*47*). More broadly, Puerto Rico has high poverty rates: in 2017, as much as 43.5% of Puerto Rico’s population was living under the poverty line, compared with 12.7% in the United States (*48*). Although TB is a disease associated with poverty (*49*), how socioeconomic factors affect TB epidemiology in Puerto Rico is uncertain, including transmission patterns, access to services, and treatment outcomes.

## Discussion

Sustained surveillance and control efforts have documented decreased rates of mortality and incidence of reported TB in Puerto Rico. In 2015, reported incidence was 1.5 cases/100,000 population, compared with the US incidence of 3.0/100,000 population. A significant increase in patient age distribution, probably resulting from a larger proportion of activated latent cases, further indicates progress. WHO has recognized Puerto Rico as 1 of 33 low-incidence jurisdictions for which TB elimination is possible by 2035 (*3*).

Three major lessons emerge from the experience of TB surveillance and control on the island. First, data collected from sustained TB surveillance have directed control efforts. Second, use of newly developed strategies and techniques has produced evidence-based practices to improve surveillance, control, and patient care. These practices include ambulatory care, use of chemoprophylaxis for latent TB, and molecular surveillance. Third, public health commitment has been crucial to TB surveillance and control efforts, as seen by dramatic declines in reported cases and mortality rates since the 1940s and 1950s.

Several areas may affect progress toward TB elimination yet remain poorly understood. Delayed diagnosis and underreporting may contribute to the current relatively low incidence of reported cases; these considerations have been historic problems for TB surveillance and control on the island (*29*–*32*). Despite improvements in care and treatment, only 50% of patients received directly observed therapy and 53% completed treatment within 1 year, indicating areas for further improvement. It remains unclear how Hurricane Maria and socioeconomic changes after the economic downturn will affect TB epidemiology, public health services, provision of care, and infrastructure. 

As demonstrated by histories of TB worldwide and in Puerto Rico (*49*), TB thrives in contexts marked by poverty. Addressing socioeconomic factors is part of the WHO framework for eliminating TB in low-incidence contexts (*3*). Although the proportion of Puerto Rico’s population living under the poverty line is 3.4 times greater than that of the rest of the United States, the effect of socioeconomic factors on TB epidemiology on the island is unclear.

Although the numbers of reported cases are becoming fewer, the ultimate goal of TB surveillance and control in Puerto Rico is TB elimination through sustained public health surveillance and control efforts, including detection, case management, and treatment success. The use of novel tools, such as molecular surveillance, electronically facilitated case consultation, and smartphone technology as an effective, low-cost measure to guide treatment adherence (*50*), has been shown to be effective for timely public health interventions and may help lead Puerto Rico to TB elimination.

Appendix Puerto Rico and United States tuberculosis mortality rates, 1932–2015.
