# Supplementary material for: Tuberculosis Surveillance and Control, Puerto Rico, 1898–2015
Source: Emerg Infect Dis. 2019 Mar;25(3):538–46. doi: 10.3201/eid2503.181157 (PMC6390739; doi:10.3201/eid2503.181157)
Supplement: Appendix — Puerto Rico and United States tuberculosis mortality rates, 1932–2015. [file 18-1157-Techapp-s1.pdf]

Article DOI: https://doi.org/10.3201/eid2503.181157

Tuberculosis Surveillance and Control, Puerto Rico, 1898–2015

Appendix

Puerto Rico and United States tuberculosis (TB) mortality rates per 100,000 population, 1932–2015 Puerto Rico, and incidence of reported TB cases, 1953–2015

Puerto Rico

Mortality Rates

1932–1993:

TB mortality data were sourced from the U.S. Centers for Disease Controls and Prevention (CDC) National Center for Health Statistics (NCHS) Vital Statistics of the United States (VSUS) (available online: https://www.cdc.gov/nchs/products/vsus.htm), and rates were calculated using Puerto Rico annual population estimates sourced from the U.S. Census Bureau, International Database.

1994–2015:

TB mortality data were sourced from the public-use mortality file from the CDC/NCHS, National Vital Statistics System, Mortality (available online: https://www.cdc.gov/nchs/data_access/vitalstatsonline.htm), and rates were calculated using Puerto Rico annual population estimates sourced from the U.S. Census Bureau, International Database.

Incidence of Reported TB Cases

Incidence of reported TB cases was calculated using the data reported in the CDC TB annual reports for cases reported during 1953–1992 and CDC Online Tuberculosis Information System (OTIS) for cases reported during 1993–2015 (available online:

https://wonder.cdc.gov/tb.html) and using Puerto Rico annual population estimates sourced from the U.S. Census Bureau, International Database.

United States

Mortality Rates

1932–1967:

TB mortality data and rates were sourced from unpublished tables from the U.S. CDC NCHS National Vital Statistics System (available online: https://www.cdc.gov/nchs/nvss/mortality_historical_data.htm).

1968–2015:

TB mortality data and rates were sourced from U.S. CDC NCHS Compressed Mortality Files (available via CDC WONDER Online Database: https://wonder.cdc.gov/mortSQL.html).

Incidence of Reported TB Cases

Incidence of reported TB cases was sourced from CDC (available online: https://www.cdc.gov/tb/statistics/reports/2017/table1.htm).
